# Task switching involves working memory: Evidence from neural representation

**DOI:** 10.3389/fpsyg.2022.1003298

**Published:** 2022-11-15

**Authors:** Yanqing Wang, Xing Zhou, Xuerui Peng, Xueping Hu

**Affiliations:** ^1^Institute of Psychology, Chinese Academy of Sciences, Beijing, China; ^2^Department of Psychology, University of Chinese Academy of Sciences, Beijing, China; ^3^Research Center of Brain and Cognitive Neuroscience, Liaoning Normal University, Dalian, China; ^4^Faculty of Psychology, Technische Universität Dresden, Dresden, Germany; ^5^School of Linguistic Science and Art, Jiangsu Normal University, Xuzhou, China; ^6^Key Laboratory of Language and Cognitive Neuroscience of Jiangsu Province, Collaborative Innovation Center for Language Ability, Xuzhou, China

**Keywords:** task switching, working memory, neural representation, brain activity pattern, conjunction analysis

## Abstract

It is generally assumed that task switching involves working memory, yet some behavioral studies question the relationship between working memory and task switching ability. This debate can be resolved by directly comparing the brain activity pattern in task switching and working memory processes. If the task switching involves working memory, the neural activity patterns evoked by such two tasks would exhibit higher similarity. Here, we employed the task switching task and working memory to investigate the characteristic of the neural representation in such two cognitive processes. A conjunction analysis showed that the bilateral superior parietal lobule (SPL), bilateral insula, bilateral middle frontal gyrus (MFG), bilateral dorsal lateral prefrontal cortex (DLPFC) and pre-supplementary motor area (pre-SMA) were commonly and significantly activated in both task switching and working memory task. Critically, we found that task switching and working memory processing elicited similar activity patterns in bilateral SPL, right insula, left MFG, left DLPFC and pre-SMA, consistent with common neural processes for both tasks. These results not only suggest that the task switching process involves working memory from the perspective of neural representation, but also provide major new insights into the neurocognitive links between task switching and working memory.

## Introduction

Performance in task switching is an important measure of cognitive flexibility. In task switching, participants randomly alternate between performances of two (or more) tasks, with an advance cue specifying the task to perform on the upcoming trial. In theory, such process presumes that multiple task sets cannot be simultaneously active, and thus successful switching requires both the deactivation of the old (now irrelevant) task set and also the re-activation of the new (now-relevant) set into working memory (Braver and Cohen, 2000; Vandierendonck, 2012). It means that task switching relies heavily on working memory to ensure control, regulation, and active maintenance of goal-relevant information (Baddeley, 2012). Indeed, it has been shown that taxing working memory makes task switching more error prone and slower (Baddeley et al., 2001; Emerson and Miyake, 2003; Bryck and Mayr, 2005; Liefooghe et al., 2005; Saeki and Saito, 2009).

More recently, some studies concerning the role of working memory in task switching are less unanimous. For instance, previous research compared task switching performance in participants with a low and a high working memory span. The results showed that high-span participants performed better than low-span participants, but working memory capacity did not interact with task switching performance (Kane et al., 2007). Also, other studies have confirmed this lack of relationship between working memory load and task switching (Logan, 2006; Kiesel et al., 2007). These findings have led researchers to call into question the theoretical accounts of task switching and claim that working memory is not involved in task switching.

Notably, the task switching performance of such researches was mainly measured through behavioral testing and indexed by reaction time switch cost, in which the mean reaction time to complete a repeat trial is subtracted from the mean reaction time to complete switch trial. The reaction time switch cost is not a precise index to reflect the difference between the experimental conditions in the task switching task. The most readily apparent problem with reaction time switch cost is that response accuracy is not taken into account. Using a score in which accuracy is completely ignored is not only problematic in group comparisons if any of the groups differ in accuracy, but also problematic in differential approaches if there are individual differences in how subjects adjust their speed and accuracy against one another (Draheim et al., 2016). For instance, most task switching studies precisely manipulate speed-accuracy tradeoffs, instructing participants to answer as quickly as possible while maintaining high accuracy. In this case, some participants may tend to maintain a high level of accuracy, resulting in a slower response. On the contrary, some hasty or impulsive participants were prone to making more mistakes on switch trials while exhibiting shorter reaction times. In addition, some participants may not be able to make appropriate speed-accuracy adjustments to the specific task being performed, while others may adjust quickly and appropriately to meet task requirements. In addition, the reaction time switch cost is the difference score and generally has low reliability, so some researchers recommend against using them in any circumstances (Edwards, 2001; Draheim et al., 2021). As a result of these issues, the use of reaction time in task switching studies can result in faulty conclusions and misguided theories.

Beyond the behavioral approach, functional neuroimaging was also used to explore specific cognitive functions. By mapping cognitive processes to the brain, neural representations of task switching and working memory processes can be directly compared, and further infer whether working memory’s involvement in task switching could provide fairly novel insights and help overcome some limitations in behavioral research. From the perspective of neural activity, the hypothesis of task switching involving working memory implies that the patterns of neural activity evoked by such two tasks would show higher similarity. It is important to note that existing studies showed that the activation of some regions in the frontal and parietal regions, such as lateral prefrontal cortex, pre-supplementary motor area (pre-SMA), anterior cingulate cortex (ACC) and superior parietal lobule (SPL), evoked by task switching and working memory is overlapping (D’Esposito et al., 2000; Marshuetz et al., 2000; Pessoa et al., 2002; Etzel et al., 2016; Loose et al., 2017; Qiao et al., 2017; Emch et al., 2019). But these common activations do not all necessarily support shared neural processing, and the underlying neural representation may not be the same. For instance, previous functional magnetic resonance imaging (fMRI) researches have revealed that physical pain and social rejection have highly overlapping activations at the gross anatomical level, but the activation patterns in response to such two processing were unrelated and dissociable (Woo et al., 2014). This result favors a functional-independence account that suggests overlapping brain activation but functionally independent neural populations are thought to be engaged within the common region.

For this, the present study utilized fMRI during such two tasks to assess whether working memory involvement in task switching. Furthermore, we performed the representational similarity analysis (RSA) to quantify the neural representational similarity of overlapping activations in task switching and working memory tasks. By analyzing and comparing distributed neural representation patterns of a set of voxels, the RSA could provide more neural activity information than conventional univariate analysis.

## Materials and methods

### Participants

The data used in this study were obtained from the OpenNeuro database with accession number of ds000030. More information about the original participant recruitment, exclusions, and study procedures can be found in the corresponding data paper (Poldrack et al., 2016). After removing participants with missing files and excessive head movement during scanning, fMRI data from a total of 106 healthy participants (53 females; mean age ± SD = 30.79 ± 8.41 years) were used in the final analysis. All participants gave written informed consent following procedures approved by the Institutional Review Boards at UCLA and the Los Angeles County Department of Mental Health.

### Task and procedure

Participants completed working memory task and task switching task in the scanner. Before scanning, participants received corresponding practice sessions to understand the stimulus–response associations and task rules.

#### Working memory task

During the working memory task, each trial began with an array of 1, 3, 5, or 7 yellow circles arranged pseudo-randomly around a fixation cross appearing on the screen for 2 s. Then a delay screen with variable length (1.5, 3, or 4.5 s) was displayed. After a delay, participants were shown a green target circle (3 s fixed response) and were asked to indicate whether that green circle was in the same position as any of the yellow circles in the initial array. There were 48 trials (12 for each array set size and 4 for each delay length), half of which were true-positive trials while the other half were true-negative trials. In the current study, the one and three sized arrays were defined as low working memory (24 trials), and the five and seven sized arrays were defined as high working memory (24 trials).

#### Task switching task

During the cued task switching task, participants were cued to perform one of two alternative tasks (shape task vs. color task) on each trial. In the shape task, cues presented included either ‘SHAPE’ or ‘S’ on trials where participants had to decide if the shape feature of the stimulus was circle or triangle. In the color task, cues presented included either ‘COLOR’ or ‘C’ on trials where participants had to decide whether the color feature of the stimulus was red or green. Participants were asked to respond as quickly and accurately as possible. There were 96 trials, including 24 switch trials and 72 repeat trials, presented in a pseudorandomized order.

### Data acquisition and preprocessing

fMRI data were collected using a Siemens Trio 3 T scanner and a Siemens 32-channel head coil. Functional images were collected using echo-planar imaging with the following parameters: repetition time (TR) = 2000 ms, echo time (TE) = 30 ms, flip angle = 90°, field of view (FOV) = 192 mm, acquisition matrix = 64 × 64, slice number = 34, slice thickness = 4 mm. The working memory task and task switching task scan lasted for 582 s (291 of images) and 416 s (208 of images), separately. T1-weighted scans were acquired with the following parameters: TR = 1900 ms, TE = 2.26 ms, FOV = 250 mm, acquisition matrix = 256 × 256, slice number = 176, and slice thickness = 1 mm.

The preprocessing of fMRI data was done using the SPM12 software^1^ on the MATLAB platform. Functional images were first temporally realigned to the middle slice for slice-timing correction, and then spatially realigned. To normalize the functional images, each subject’s structural brain image was co-registered to the mean functional image and was subsequently segmented. The parameters obtained during segmentation were used to normalize each subject’s functional image onto the Montreal Neurological Institute space (voxel size, 2 mm^3^). The normalized data were smoothed using a 6 mm full-width half-maximum Gaussian kernel.

### Univariate activation

A general linear model approach was used to estimate each task event. The regressors of task switching task included repeat trials and switch trials; The regressors of working memory task include one sized array, three sized array, five sized array and seven sized array. For each trial of task switching task, a stick function with 0 duration was used from the onset of the cue. For each trial of working memory task, a single boxcar function was used from the onset of the encoding period to the end of the response period. In addition, six head-motion parameters were included in the model as regressors of non-interest. All regressors were convolved with the canonical hemodynamic response function. A high-pass filter of 1/128 Hz was implemented to remove low-frequency drift from the time series. After model estimation, the contrast between the switch and repeat conditions was defined for task switching task; the contrast between the high working memory (five and seven sized arrays) and low working memory (one and three sized arrays) was defined for working memory task. These first-level contrasts were submitted to second-level analysis by using one-sample *t*-test.

### Neural representational similarity in overlapping activation

In order to find regions with similar neural representation, a conjunction analysis was first performed to identify the overlapping activation regions across the task switching and working memory tasks with the voxel-wise threshold of *p* < 0.05 (family-wise error corrected, cluster size >20).

Then, we used RSA to quantify the neural representational similarity in overlapping activation in two tasks (Kriegeskorte et al., 2008). For each region of interest (ROI), we combined the response amplitudes (i.e., contrast map from the second-level analysis) over all of the included ROI voxels to form activity vectors for task switching task and working memory task, respectively. Each vector can be understood as the distributed neural representation of task switching task or working memory task across the included ROI voxels. To estimate the similarity between the response patterns corresponding to task switching task and working memory task, we calculated the Pearson correlation between activity vectors corresponding to these two tasks.

## Results

**Figure 1:**
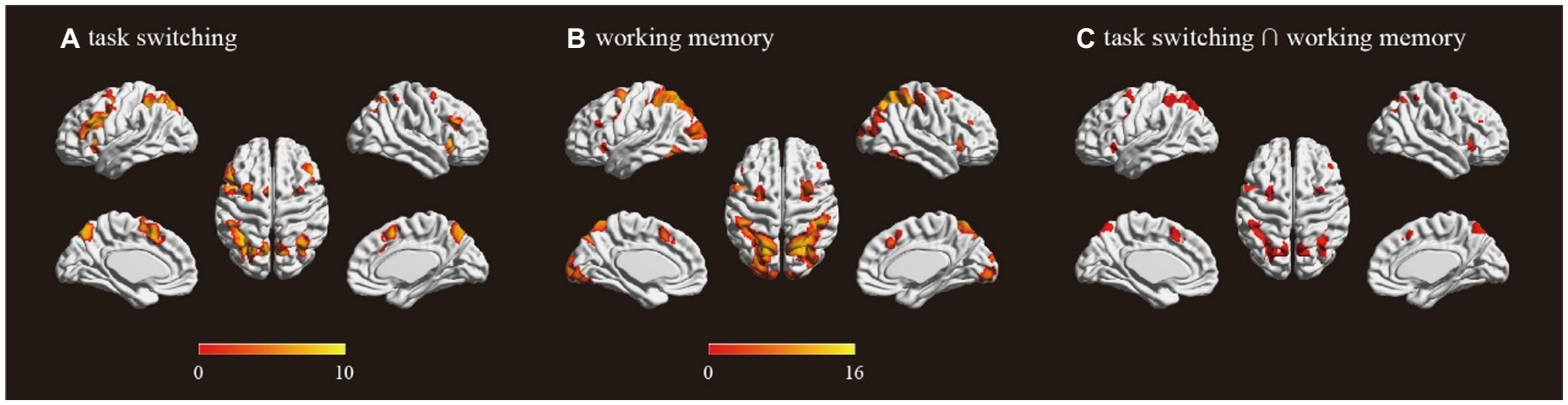
Activations for **(A)** task switching (switch trial > repeat trial); **(B)** working memory (high working memory > low working memory); **(C)** overlap of task switching and working memory. Color bar shows a scale of the *t* values and all results were corrected at *p* < 0.05 by FWE (Family-wise error).

The univariate analysis revealed that the task switching processing was associated with engagement in frontoparietal regions including bilateral SPL, bilateral insula, bilateral middle frontal gyrus (MFG), bilateral dorsal lateral prefrontal cortex (DLPFC), pre-SMA and ACC (Figure 1A; Table 1). The working memory maintenance was associated with activation in bilateral SPL, bilateral insula, bilateral MFG, bilateral DLPFC pre-SMA and occipital lobe (Figure 1B; Table 1). The conjunction analysis confirmed the overlap between the task switching and working memory maintenance-related activation in bilateral SPL, bilateral insula, bilateral MFG, left DLPFC and pre-SMA (Figure 1C; Table 2).

**Table 1:** Brain regions associated with task switching and working memory processes.

Region	Cluster size	Montreal Neurological Institute (MNI) coordinates			*t*	*p*
		x	y	z		
Task switching task						
Superior parietal lobule	2,607	−30	−60	46	9.54	<0.001
		−32	−48	42	9.25	<0.001
		−26	−68	46	8.11	<0.001
Dorsal lateral prefrontal cortex	1,376	−44	10	28	9.09	<0.001
		−46	2	30	8.99	<0.001
		−50	2	40	8.02	<0.001
Supplementary motor area	577	0	14	48	8.58	<0.001
		−8	2	64	6.34	=0.001
Insula	137	−30	20	0	8.44	<0.001
Insula	188	32	20	2	8.16	<0.001
		40	18	−4	5.99	=0.004
Superior parietal lobule	466	36	−66	48	7.58	<0.001
		34	−58	50	7.38	<0.001
		30	−54	42	6.94	<0.001
Dorsal lateral prefrontal cortex	116	50	28	30	7.09	<0.001
		36	28	28	5.74	=0.013
Middle frontal gyrus	276	−26	6	54	7.02	<0.001
		−28	−4	48	6.36	=0.001
		−28	4	64	6.19	=0.002
Middle frontal gyrus	69	32	2	56	6.6	<0.001
Working memory task						
Superior parietal lobule	5,039	22	−64	56	15.3	<0.001
		28	−66	40	13.5	<0.001
		42	−36	52	13.45	<0.001
Superior parietal lobule	7,544	−20	−64	58	15.07	<0.001
		−24	−68	36	11.86	<0.001
		−40	−38	40	11.12	<0.001
Inferior Temporal gyrus	364	52	−56	−14	10.3	<0.001
Middle frontal gyrus	475	−26	−2	58	10	<0.001
Middle frontal gyrus	652	30	0	60	9.74	<0.001
Dorsal lateral prefrontal cortex	410	−48	6	30	9.13	<0.001
Supplementary motor area	263	−2	16	48	7.27	<0.001
		6	26	36	6.73	<0.001
Insula	83	30	26	−4	7	<0.001
Dorsal lateral prefrontal cortex	39	46	6	24	6.78	<0.001
Occipital lobe	50	16	−78	−16	6.53	<0.001
Insula	34	−32	22	−4	6.23	=0.001
Middle frontal gyrus	29	48	34	30	6.02	=0.004

**Table 2:** Neural representational similarity in overlapping activations.

Region	Cluster size	*r*	*p*
right SPL	360	−0.20	<0.001
right MFG	66	0.02	=0.89
right insula	55	0.48	<0.001
left SPL	1,431	−0.15	<0.001
left MFG	143	−0.18	=0.03
left insula	25	−0.02	=0.93
left DLPFC	333	0.28	<0.001
pre-SMA	180	0.48	<0.001

SPL, Superior parietal lobule; MFG, Middle frontal gyrus; DLPFC, Dorsal lateral prefrontal cortex; pre-SMA, Supplementary motor area.

Intuitively, if the neural overlap of activity within these regions serving task switching and working memory reflects cognitive processes common to both tasks. Then the brain activities underlying task switching processing and working memory should also show representational similarity in their respective spatial distribution within the overlapping clusters (Thibault et al., 2021). The representational similarity analysis revealed that the activation pattern of bilateral SPL (left SPL: *r* = −0.15, *p* < 0.001; right SPL: *r* = −0.20, *p* < 0.001), right insula (*r* = 0.48, *p* < 0.001), left MFG (*r* = −0.18, *p* = 0.03), left DLPFC (*r* = 0.28, *p* < 0.001) and pre-SMA (*r* = 0.48, *p* < 0.001) exhibited significantly similar neural representation across such two task (Table 2), indicating the existence of shared cognitive processing between the task switching and working memory. Obviously, the shared cognitive processing reflects working memory function to ensure control, regulation, and active maintenance of goal-relevant information.

## Discussion

There is no general agreement on the involvement of working memory processes in task switching. The present study combined fMRI with representational similarity analyses to provide neural support for the assumption that task switching processing involves working memory. First, task switching and working memory rely on brain activity of overlapped frontoparietal regions, particularly in bilateral SPL, bilateral insula, bilateral MFG, bilateral DLPFC and pre-SMA. Second, task switching and working memory processing elicited similar activity patterns in bilateral SPL, right insula, left MFG, left DLPFC, and pre-SMA, consistent with common neural processes for both tasks.

The results of our univariate analysis identification of brain regions involved in processing the task switching closely replicate previous findings and thus confirm that the SPL, insula, MFG, DLPFC, pre-SMA and ACC support switch between two tasks. In order to switch between two tasks, the relevant task set must be retrieved from long-term memory by means of executive processes and subsequently maintained in working memory (Mayr and Kliegl, 2000; Rubinstein et al., 2001). Maintaining the information representation of the task goal in working memory helps to keep attention focused on the task at hand (Kessler and Meiran, 2008; Frenken and Berti, 2018). Accordingly, such regions were separately involved in executive processes and working memory during switching between two tasks. Our results not only found that the task switching and working memory showed overlapping activation in the bilateral SPL, bilateral insula, bilateral MFG, left DLPFC, and pre-SMA, but also particularly found that the activity of bilateral SPL, right insula, left MFG, left DLPFC, and pre-SMA during task switching processing showed similar activity patterns with working memory processing. This suggests that the switch between two tasks involves working memory processes. When it comes to the performance of task switching tasks, working memory system ensures control, regulation, and active maintenance of goal-relevant information (Frenken and Berti, 2018).

Moreover, the activation of DLPFC and parietal region was observed with different types of task cues in task switching. For example, Bode and Haynes (2009) used multi-voxel pattern analysis to successfully decode task-related signals in DLPFC and parietal region during a task in which participants were cued with one of two simple stimulus–response rules to apply to an upcoming target stimulus (Bode and Haynes, 2009). These results suggest that the working memory function, i.e., configuring and maintaining the different task settings needed to perform an upcoming task, is essential for task switching and highlights the significant role of DLPFC and parietal region in supporting such function. Our results corroborated these findings and took a step further with the help of RSA to demonstrate that the role played by DLPFC and parietal region in task switching is working memory processing.

Take into consideration that task switching is often thought to require changing task (or stimulus–response mapping) rules, it is necessary to encode the associations between relevant stimuli and responses to perform a task. The pre-SMA has been repeatedly reported in previous neuroimaging and Transcranial Magnetic Stimulation literature on task switching (Rushworth et al., 2002), with the latter suggesting a critical role for this region in transiently selecting between specific response sets rather than in switching *per se*. In addition, using cue target types of paradigms found that the pre-SMA was activated after the target presentation, which was independently of the type of task, indicating that it has a role in implementing the correct stimulus–response mappings (Chiu and Yantis, 2009). Our results revealed that the pre-SMA during task switching processes showed similar activity patterns with working memory. This result suggests that the retrieved specific response of relevant stimuli from long-term memory and maintained in working memory is important to successful performance during cued task switching.

In conclusion, this study provides evidence that task switching processing elicits similar neural activity patterns with working memory processing, indicating common neural processes for such two tasks. To our knowledge, this is the first study to combine cued task switching and working memory tasks with functional magnetic resonance imaging and harnessed representational similarity analysis to assess whether working memory involvement in task switching. Our findings provide a better understanding of the processing mechanism of task switching from the perspective of neural representation, highlighting the involvement of working memory in task switching.

## Data availability statement

The original contributions presented in the study are included in the article/Supplementary material, further inquiries can be directed to the corresponding author.

## Ethics statement

The studies involving human participants were reviewed and approved by Institutional Review Boards at UCLA and the Los Angeles County Department of Mental Health. The patients/participants provided their written informed consent to participate in this study.

## Author contributions

YW: data curation, formal analysis, validation, investigation, and writing—original draft. XZ: writing—review and editing. XP: writing—review and editing. XH: supervision, funding acquisition, methodology, and writing—review and editing.

## Funding

This research was funded by the National Natural Science Foundation of China (31900750), and the Natural Science Research Foundation of Jiangsu Normal University (18XLRX011).

## Conflict of interest

The authors declare that the research was conducted in the absence of any commercial or financial relationships that could be construed as a potential conflict of interest.

## Publisher’s note

All claims expressed in this article are solely those of the authors and do not necessarily represent those of their affiliated organizations, or those of the publisher, the editors and the reviewers. Any product that may be evaluated in this article, or claim that may be made by its manufacturer, is not guaranteed or endorsed by the publisher.

## Footnotes

^1^ https://www.fil.ion.ucl.ac.uk/spm12/
